# Optical Properties and Antimicrobial Activity of Si/PVP Hybrid Material Combined with Antibiotics

**DOI:** 10.3390/molecules29225322

**Published:** 2024-11-12

**Authors:** Lilia Yordanova, Yoanna Kostova, Elitsa Pavlova, Albena Bachvarova-Nedelcheva, Iliana Ivanova, Elena Nenova

**Affiliations:** 1 Faculty of Biology, Sofia University “St. Kliment Ohridski”, 8 Dragan Tsankov Blvd., 1164 Sofia, Bulgaria; lilia_petrova3@abv.bg (L.Y.); iaivanova@biofac.uni-sofia.bg (I.I.); nenova@uni-sofia.bg (E.N.); 2 Institute of Metal Science, Equipment and Technologies with Hydro- and Aerodynamics Centre “Acad. A. Balevski”, Bulgarian Academy of Sciences, Shipchenski Prohod Str., 67, 1574 Sofia, Bulgaria; y_kostova@ims.bas.bg; 3 Faculty of Physics, Sofia University “St. Kliment Ohridski”, 5 James Boucher Blvd., 1164 Sofia, Bulgaria; elli_pavlova@abv.bg; 4 Institute of General and Inorganic Chemistry, Bulgarian Academy of Sciences, Acad. G. Bonchev Str., Bl. 11, 1113 Sofia, Bulgaria

**Keywords:** hybrid, optical properties, antibacterial properties, antibiotics, prooxidant properties, environmental toxicity control

## Abstract

Silica–poly (vinylpyrrolidone) hybrid material was prepared using the sol–gel method. Tetramethyl ortosilane (TMOS) was used as a silica precursor. XRD analysis established that the as-prepared material is amorphous. The morphological structure of the final product was determined by the incorporated PVP. The UV–Vis analysis showed that the obtained hybrid exhibited absorption in the ultraviolet range. The antimicrobial activity of the SiO_2_/15PVP hybrid material was tested on *Staphylococcus epidermidis* ATCC 14990, *Salmonella typhimurium* ATCC BAA-2162, *Candida albicans*, and *Saccharomyces cerevisiae* in combination with the following antibiotics: Vancomycin for Gram-positive bacteria, Ciprofloxacin for Gram-negative bacteria, and Nystatin for yeast. The results confirmed a concentration-dependent synergistic effect of the antibiotic in combination with the TM15/PVP hybrid particles, especially at their highest concentration of 100 mg/mL on Gram-positive bacteria and for the Gram-negative *Salmonella*. On *Candida albicans* ATCC 18804 and *Saccharomyces cerevisiae* CCY 21-6-3, the effect was synergistic again, and a fungicidal effect was observed at 6.25 and 1.50 mg/mL for the antibiotic concentration and concentrations of hybrid material at 100 mg/mL. The toxicity on *Daphnia magna* was also tested. The registered prooxidant activity of SiO_2_/15PVP shows possible applications at very low concentrations. The obtained results demonstrate the possibility of clinical implementations of the newly synthesized hybrid material.

## 1. Introduction

One of the important extensions of the sol–gel process is the preparation of a group of transparent amorphous materials consisting of an organic polymer and a metal oxide such as silica gel [1]. These hybrid materials are constituted by an amorphous network of inorganic and organic phases with strong interface interactions, typically through coupling agent molecules. Therefore, by choosing the appropriate phases, hybrid materials with desirable properties can be designed for multiple purposes [2,3].

It is recognized that nanoparticles, with their unique properties, such as their high surface area to volume ratio, quantum effects, enhanced reactivity, antibacterial activity, oxidation resistance, and high thermal conductivity, are leading the way in the world of modern science and technology toward a true revolution in various fields, including medicine, electronics, and environmental protection [3]. Among the wide range of nanomaterials, mesoporous nanoparticles stand out with exceptional properties such as biocompatibility, easy modification, and the ability to accommodate drug molecules [4]. Therefore, these kinds of materials are excellent candidates for the growing problem of bacterial antibiotic resistance worldwide [5].

Generally, silica nanomaterials are still of great interest to modern science and biomedicine due to their excellent properties that enable numerous biological applications [6]. Inorganic silica nanoparticles have a high surface-to-volume ratio and exhibit tissue compatibility and stability in biological macro-organisms. Due to its pronounced biocompatibility, inertness, low toxicity, resistance to acidic conditions, and thermal resistance, silica has the ability to enter into a variety of biochemical interactions due to its ability to form covalent bonds with macromolecules, such as nucleic acids, antibodies, fluorescent molecules, thanks to the silanol groups in its structure [7]. Silica nanoparticles are highly prevalent in nanotechnology because their production is an easy and cheap process. They are also used as biocompatible pharmaceutical additives. Numerous studies have been developed that prove the low toxicity and high bactericidal efficiency of mesoporous silica nanoparticles whose surface is modified with organic polymers, for example, PVP, and whose interior is loaded with antibiotics [8].

It is well known that the generation of reactive oxygen species (ROS) is one of the main toxicity mechanisms by which nanoparticles inhibit bacterial growth. The generation of ROS is an important biomarker regarding the redox properties of substances. By following the kinetics of the generation of free radicals and ROS, the properties of newly synthesized materials can be evaluated as well as their effects on the cascade of reactions causing the formation and accumulation of antibacterial free radicals and ROS [9,10].

Chemiluminescence analysis is a rapid and sensitive method for such studies. It is applied to follow the dynamics of free radical and ROS generation and to determine the prooxidant/antioxidant activity of various materials. The responses are recorded in the range of 480–580 nm and can be used to estimate the quantum yield [10,11,12]. The redox activity of the newly synthesized material was tested in Fenton’s model chemical reaction system, generating free radicals and ROS, at 7.4/37 °C (physiological), using the activated chemiluminescence method [11]. In general, the higher the signal, the more ROS are formed and the pro-oxidant effect is demonstrated. On the contrary, if the signal is below the control level, antioxidant activity can be found.

The combination of nanoparticles (NPs) with antibiotics may improve the antibacterial effect through additive or synergistic antibacterial effects by chemically or physically weakening the bacteria [13,14]. It has been shown that such mechanisms weaken bacteria by the creation of reactive oxygen species, antibacterial ions, or elevated temperatures [15]. The synergistic effects between metal and metal oxide NPs (MNPs) and commercial antimicrobial drugs have been studied for several years [14]. Most of the synergistic studies focus on silver nanoparticles (AgNPs), gold (Au), copper (Cu), copper oxide (CuO), copper sulfide (CuS), iron (Fe), iron oxide (Fe_3_O_4_/Fe_2_O_3_), zinc, zinc oxide (ZnO), and platinum (Pt). MNPs have been combined with several antibiotic, antifungal, and antiviral agents, but a high number of compounds still remain unexplored. However, their mechanism of action is still not completely understood [16]. Recently, it has been reported that the conjugation of MNPs with other antimicrobial compounds may enhance their effectiveness [14]. Some authors have found new approaches in the fight against pathogens including the revival of old antibiotics, to overcome the current drug resistance emergency [13,17]. Drug combination is a common strategy in clinical practice, and its therapeutic success has been found in the treatment of acquired immunodeficiency syndrome (AIDS), cancer, cardiovascular disease, and microbial infections [14].

The present work is a continuation of our previous investigations on the synthesis and antibacterial properties of sol–gel-derived hybrid silica–poly (vinylpyrrolidone) materials [8]. This study particularly demonstrates that it is possible to find effective combinations of nanostructures and conventional antibiotics to prevent the formation of bacterial biofilms and bacterial resistance. It was discovered that in combination, nanoparticles and antibiotics exert significant bactericidal activity against both susceptible and already resistant strains. The synergistic antimicrobial effect that allows for the inhibition of bacterial growth, using low concentrations of both the antibiotic and the nanoparticles, emphasizes the novelty of the paper.

## 2. Results and Discussion

### 2.1. XRD and SEM Observations

**Figure 1:**
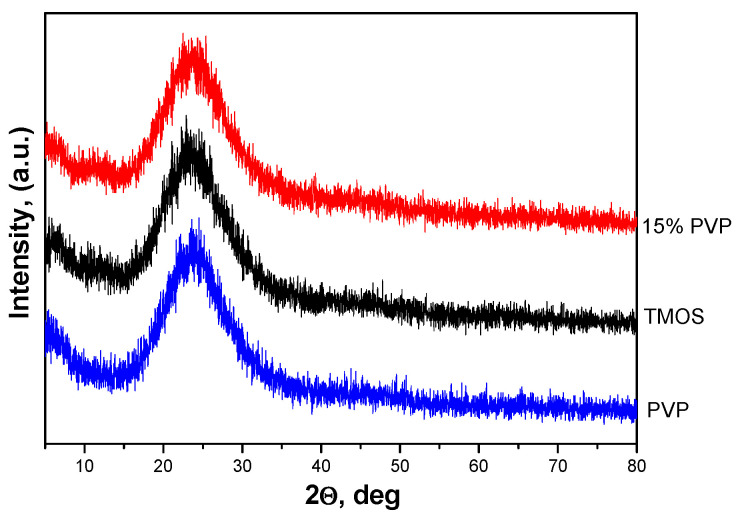
XRD patterns of SiO_2_/15PVP, TMOS, and PVP.

Figure 1 displays the X-ray diffraction patterns of samples with 15% PVP content as well as pure TMOS and PVP samples. The figure illustrates that all gels are amorphous. This result correlates well with those obtained in our previous investigations [8].

**Figure 2:**
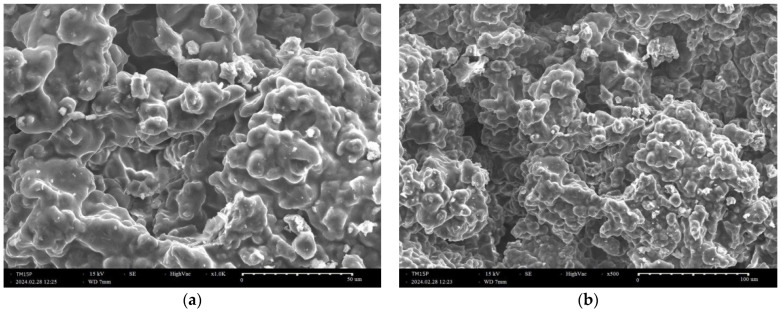
SEM micrographs of a sample with 15% PVP at different magnifications (**a**,**b**).

SEM images of the investigated gel are shown in Figure 2a,b. The analysis of the morphology of the SiO_2_/15PVP sol–gel derived powder showed clusters of agglomerated particles. The same images also exhibited the presence of particles with irregular shapes. However, other samples with larger quantities of PVP are characterized by such particle agglomerates [18]. This is due to the high linking ability of PVP. Some authors [18] have stated that during the formation of the silica core after the hydrolysis and polycondensation of TEOS and PVP, molecules interact with the silica particles via electrostatic and intermolecular hydrogen bonds between the OH groups of silica and C=O groups of PVP. Consequently, at higher PVP (>10 mol%) concentrations, large particle clusters could be formed during the sol–gel process. This influence of PVP on morphology is in accordance with our previous study [8].

### 2.2. UV–Vis Characterization

**Figure 3:**
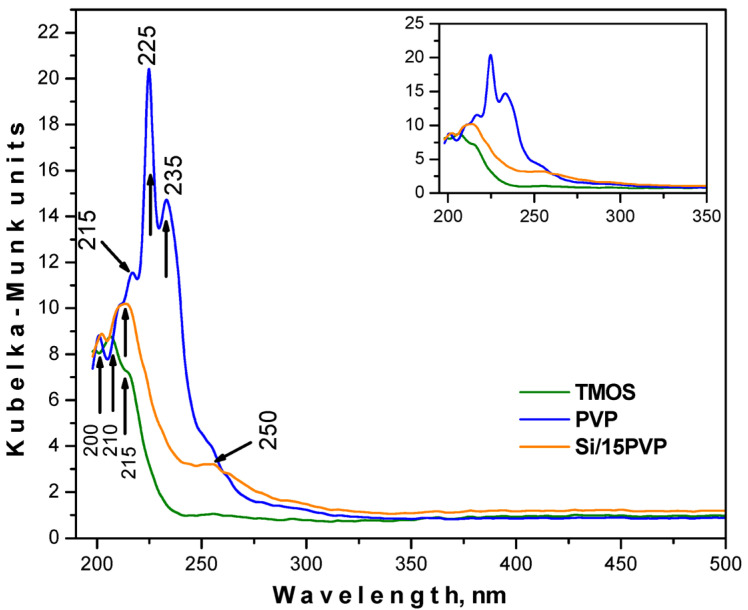
UV–Vis spectra for the pure TMOS, PVP, and SiO_2_/15PVP hybrid. The inset shows the spectra in the range of 200–350 nm.

Aiming to analyze the optical properties of the produced gel, UV–Vis spectra were applied in the range from 190 to 500 nm for pure TMOS, PVP, and the obtained hybrid material (Figure 3). As can be seen from the figure, all spectra exhibited bands in the UV region only. Several absorption peaks (215, 225, and 235 nm) are characteristic of pure PVP, with that at 225 nm being the most intensive one. The increased absorption of PVP in the UV areas is probably due to the type of transitions n → π* occurring between pairs of free charge carriers derived from oxygen atoms and vacant states [19]. TMOS showed strong absorption for ultraviolet wavelengths below 230 nm, which is consistent with the results presented by Xu et al. [20]. This phenomenon can be caused by defects in the surface structure of the produced nanoparticles, resulting in the formation of an incomplete four-wall network of Si–O–Si, which is characterized by local optical activity only [20]. The spectra of the obtained hybrid SiO_2_/15PVP are characterized by a sharp absorption edge at 220 nm. In addition, an increase in the absorption of the composite in comparison to the pure TMOS was recorded. Using the registered UV–Vis spectra, the cut-off and energy gaps (Eg) values are shown in Table 1. The calculated Eg values for pure amorphous precursors TMOS and PVP are 5.43 and 4.63 eV, respectively, which corresponds well with the data obtained by other authors [19,21]. It has to be noted that the Eg value of pure TMOS is usually found to be above 9 eV, as reported by Weinberg et al. [21]. Nowadays, the production of SiO_2_ nanostructures with lower Eg values can significantly affect the performance improvement of electronic components used in production processes that employ silica [19]. Looking back to Table 1, it can be seen that the band gap of the hybrid is 5.22 eV, with it shifting to lower energy waves, probably due to the nanometric size of the obtained gel. Bearing in mind that the absorption of the hybrid increases in comparison to the pure TMOS, it might be suggested that the obtained material could be used as an efficient absorber of ultraviolet radiation.

### 2.3. AFM Investigations

**Figure 4:**
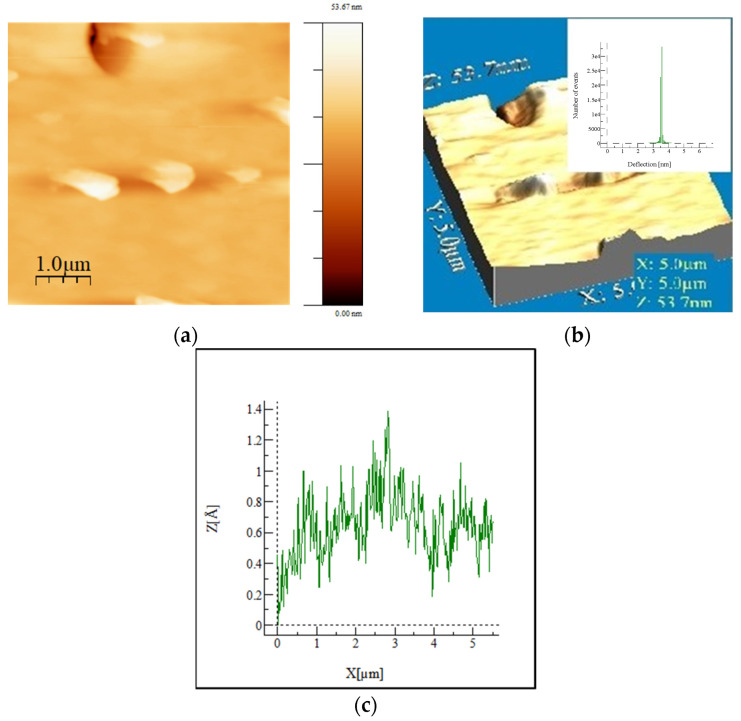
AFM images of the investigated SiO_2_/15PVP hybrid: 2D (**a**) and 3D (**b**) surface topography and roughness profile (**c**) of the material.

The AFM images in Figure 4 show the morphology of the micro- and nanostructured surface of the hybrid SiO_2_/15PVP material. The scanned area has dimensions of 5 × 5 μm^2^ and a scanning speed of 10 kH. From Figure 4a, it can be seen that the sample has a relatively smooth surface with negligible irregularities but significant homogeneity. Figure 4b is a three-dimensional topographic map from which the main parameters of the AFM analysis are determined. The inset in Figure 4b exhibits the hysteresis of the height distribution profiles of surfaces from which the mean square roughness (RMS) is determined. It represents the standard deviation of the height value in the selected region, which is 0.089 nm, and the calculated Ra (roughness average) for the scanned area is 0.055 nm. The surface height distribution profile (Figure 4c) obtained from the 3D image shows that the surface morphology of the inorganic–organic hybrid SiO_2_/15PVP material is homogeneous and structured by minor irregularities with relatively low height. Other AFM studies of sol–gel hybrid materials show a good homogeneous continuous structure, as well as certain aspects of the morphology of the surface depending on the chemical composition and percentage of the organic additive [22,23].

### 2.4. Antibacterial Properties

In our research, we tested the effect of SiO_2_/15PVP hybrid nanomaterials alone and in combination with popular antibiotics: Vancomycin against the Gram-positive bacteria *Staphylococcus epidermidis* ATCC 14990, Ciprofloxacin against the Gram-negative bacteria *Salmonella typhimurium* ATCC BAA-2162, and Nystatin against two eukaryotic cells—the yeasts *Candida albicans* ATCC 18804 *and Saccharomyces cerevisiae* CCY 21-6-3.

**Figure 5:**
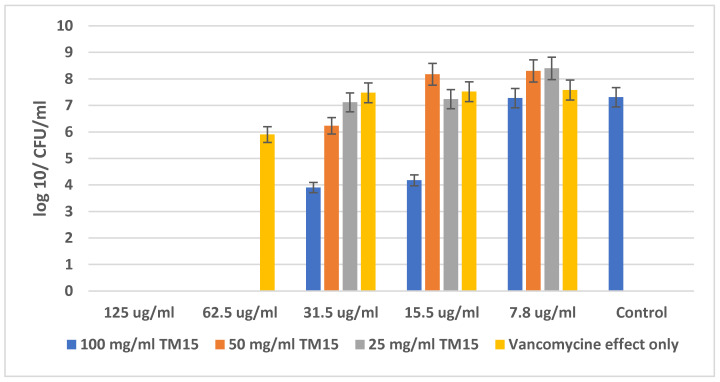
Effect of SiO_2_/15PVP in combination with Vancomycin against *Staphylococcus epidermidis* ATCC 14990 at 24 h of treatment.

Figure 5 shows the amount (in CFU/mL) of *Staphylococcus epidermidis* ATCC 14990 at different concentrations of SiO_2_/15PVP (100 mg/mL, 50 mg/mL, and 25 mg/mL) in combination with the antibiotic Vancomycin and compared to the effect of the antibiotic alone. The MBC of the single action of the antibiotic Vancomycin against *Staphylococcus epidermidis* was determined—125 µg/mL, while in the combination of Vancomycin with the highest concentration of SiO_2_/15PVP (100 mg/mL), the MBC already decreased to only 62.5 µg/mL for the antibiotic concentration. The results in Figure 5 show that the minimum inhibition concentration (MIC) of single Vancomycin is 62.5 µg/mL, and the minimum inhibition concentration of 100 mg/mL SiO_2_/15PVP in combination with Vancomycin is 31.5 µg/mL.

**Figure 6:**
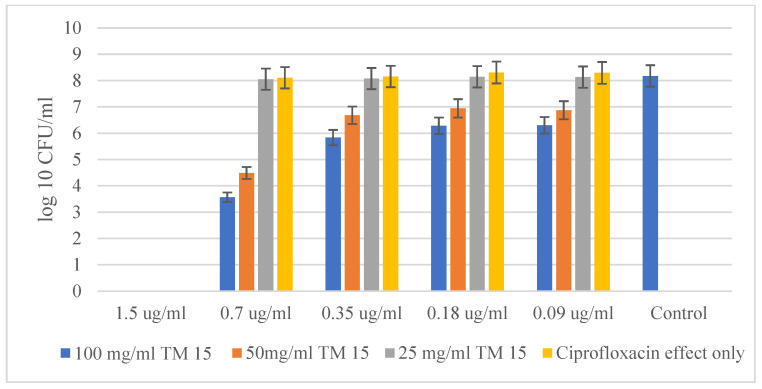
SiO_2_/15PVP in combination with Ciprofloxacin against *Salmonella typhimurium* ATCC BAA-2162 at 24 h of the experiment.

The experiments with the Gram-negative bacteria *Salmonella typhimurium* ATCC BAA-2162 and the antibiotic Ciprofloxacin showed similar results in the resistance of the bacteria against the antibiotic alone and in the combination of Ciprofloxacin with the highest concentration of the hybrid. The obtained results for *Salmonella typhimurium* are quite similar to those obtained for *Staphylococcus epidermidis* (Figure 6). The chart does not show a big difference in MBC between the independent action of the antibiotic and when it is combined with the highest concentration of the hybrid material SiO_2_/15PVP (100 mg/mL). The MBC of the antibiotic acting alone against *Salmonella typhimurium* ATCC BAA-2162 was found to be 1.5 µg/mL, which is the same value as the effect of Ciprofloxacin in combination with SiO_2_/15PVP. Interestingly, the data from the graph confirm the significant synergistic effect of the antibiotic in combination with SiO_2_/15PVP, especially at their highest concentration of 100 mg/mL. As the minimum inhibitory concentration (MIC) of Ciprofloxacin in combination with the hybrid nanomaterials was 0.7 µg/mL, it is significantly lower than that of the antibiotic acting alone. The difference in the microbial quantity of the antibiotic alone and the combination of antimicrobial agents is almost four times more.

### 2.5. Antifungal Effects

**Figure 7:**
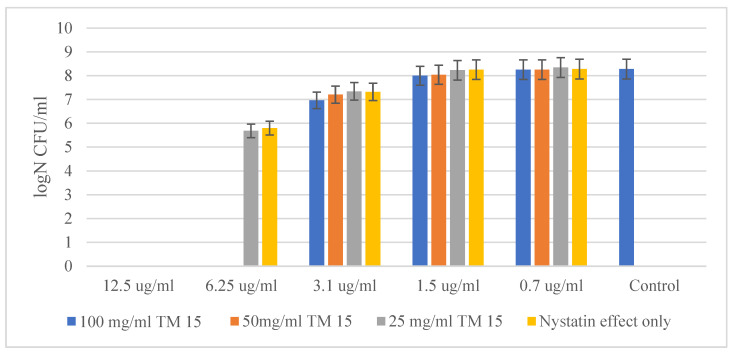
Effect of SiO_2_/15PVP in combination with Nystatin against *Candida albicans* ATCC 18804 at 24 h of treatment.

As mentioned above, SiO_2_/15PVP hybrid nanomaterials were also tested in combination with the antifungal agent Nystatin against two eukaryotic cells. Of the two yeast cells tested, *Candida albicans* ATCC 18804 showed higher resistance to the concentration of antibiotics and the hybrid material. Here, the MBC of Nystatin alone was 12.5 µg/mL, while through a synergistic effect with the new hybrid material SiO_2_/15PVP at a concentration of 100 mg/mL, the MBC decreased to 6.25 µg/mL, as shown in Figure 7.

**Figure 8:**
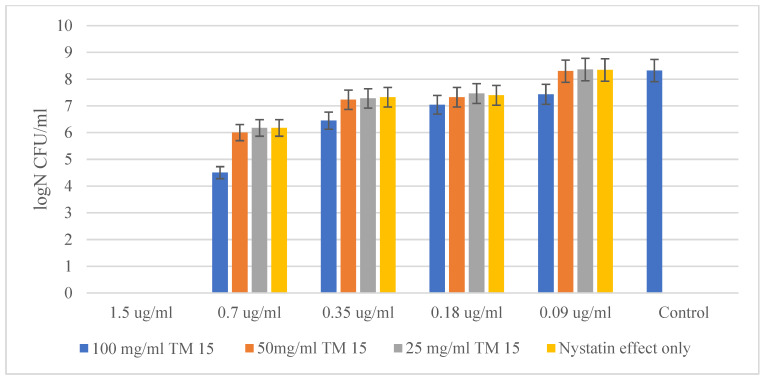
SiO_2_/15PVP in combination with Nystatin against *Saccharomyces cerevisiae* CCY 21-6-3 at 24 h of the experiment.

The other yeast tested showed quite similar results in its suppression by Nystatin and the hybrid material. However, *Saccharomyces cerevisiae* CCY 21-6-3 turned out to be a much more sensitive microorganism. Figure 8 shows a similar MBC of Nystatin acting alone and in combination with 100 mg/mL of the hybrid material. The MBC of Nystatin against *Saccharomyces cerevisiae* was established to be 1.5 µg/mL, while for the combination of Nystatin and the highest concentration (100 mg/mL) of SiO_2_/15PVP, the MIC was established to be 0.7 µg/mL. Microbial growth was inhibited by almost 10,000 times.

### 2.6. Luminescence Tests for SiO_2_/15PVP

**Figure 9:**
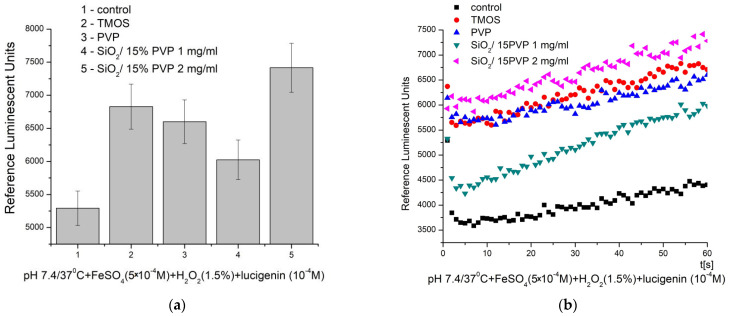
Chemiluminescence induced by ·OH and ·OOH radicals at pH 7.4/37 °C and the effect of the materials: (**a**) maximum effects and (**b**) Fenton’s reaction over time (*p* ≤ 0.05).

The results of the chemiluminescence assay and the potential of the tested material to generate ROS and present antibacterial properties are presented in Figure 9.

At pH 7.4/37 °C (physiological), the kinetic curves showed a gradual increase in the signal over time, indicating the generation of ROS in both the control and test reactions. The control reaction had the lowest signal, with a maximum of around 5300 RLU. In all other cases, the recorded signal was higher than the control, indicating the stimulated generation of ·OH and ·OOH radicals for the newly synthesized material and its precursors. The observed effects were as follows: TMOS—29%, PVP—25%, SiO_2_/15PVP (1 mg/mL)—14%, and SiO_2_/15PVP (2 mg/mL)—40%. These results demonstrate a significant, nearly threefold increase in the effect of the newly synthesized material at low, borderline application concentrations—2 in comparison to 1 mg/mL. The newly synthesized SiO_2_/15PVP compound showed a significant pro-oxidant effect that persists over time. In comparison, our earlier results, described in [8], showed a similar prooxidant effect for the SiO_2_/20PVP material in the same reaction and conditions at a significantly higher final concentration (25 mg/mL). The differences in the control reaction signal are due to the extreme sensitivity of the method, which is why controls are prepared for each evaluation.

### 2.7. Results on Daphnia Magna Toxicity Testing

**Figure 10:**
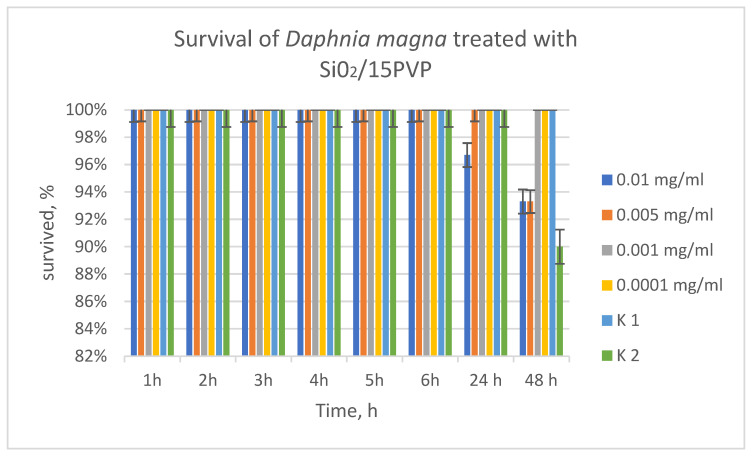
TM/15PVP without antibiotic influence on *Daphnia magna*.

*Daphnia* are extremely sensitive organisms in the aquatic environment, which allows conclusions to be drawn from the obtained results about the influence of SiO_2_/15PVP on other aquatic organisms as well. Behavioral results of *Daphnia* showed 100% survival in the acute toxicity test at 0.001 and 0.0001 mg/mL concentration. At the highest concentration (0.01 mg/mL) at 24 h and 48 h, 97% of *Daphnia* survived. At the second concentration (0.005 mg/mL) up to the twenty-fourth day, survival was 100%, and at 48 h, 97% survival of *Daphnia* was observed. Compared to studies performed on the effect of PVP 20% on *D. magna* [7], the present results showed that PVP 15% was even better tolerated by *Daphnia*, close to 100%. Therefore, these concentrations of the substance used (PVP 15%) can be considered harmless and can be discharged for whatever reason into surface waters without harm to organisms (Figure 10).

### 2.8. Analysis of the Results

The above-presented experimental results confirm the trend that silica-containing materials possess a bacteriostatic effect [8,24,25]. These data correspond well with our previous data [8], but the present survey expands the investigations with other bacterial strains (*Staphylococcus epidermidis* ATCC 14990 and *Salmonella typhimurium* ATCC BAA-2162), as well as different fungi (*Candida albicans* ATCC 18804 and *Saccharomyces cerevisiae* CCY 21-6-3). Based on the obtained results, it could be concluded that all of the bacteria, both Gram-positive and Gram-negative, are sensitive to the presence of the hybrid material together with appropriate antibiotics. Once again, it has been demonstrated that the synergistic effect of the hybrid with antibiotics is specific to the tested bacteria. The antibacterial activity of the silica hybrid is evidently activated upon functionalization with relatively low concentrations of the antibiotics Ciprofloxacin, Vancomycin, and Nystatin. Obviously, the combination of the antibiotics and the SiO_2_/15PVP material strongly affects the antibacterial properties of the synthesized material. The synergistic effects of Si/PVP with antibiotics against microorganisms have been described by several authors in the literature [14,26]. Generally, they can be explained by the formation of chelation bonds, which are considered one of the most important interactions as they increase the concentration of antimicrobial agents on the cell membrane. Perhaps, the synergistic effects of Si/PVP with antibiotics against microorganisms could be explained by reactive oxygen species (ROS), which are formed in lower concentrations. Additional confirmation for the good antibacterial activity of the synthesized SiO_2_/15PVP compound is the significant pro-oxidant effect that persists over time. These results provoke our scientific interest to continue these experiments, and future studies will focus on the investigation of the kind of prepared hybrid and the influence of the composition of the antibacterial properties. Such information could shed new light on the possibility of using these materials for several biomedical applications including drug delivery.

## 3. Experiment

### 3.1. Materials and Preparation of Silica–Polyvinylpyrrolidone (SiO_2_-PVP) Hybrids

The gels were prepared using a combination of the alkoxide tetramethyl orthosilane (TMOS), Sigma Aldrich Chemical (St. Louis, MO, USA), and polyvynylpyrrolidon K25-(PVP), Fluka AG. (Buchs, Switzerland). The preparation procedure is described in detail below. The following precursors, tetramethyl orthosilicate {TMOS, Si(OCH_3_)_4_}, distilled H_2_O, and 0.1 M HCl in a molar ratio of 4:1:1, were mixed and stirred together. PVP at 15 wt% was added with the aim of obtaining a hybrid material with improved properties. It was dissolved in ethanol and added dropwise to the stirred solution. Stirring was continued until the mixture became clear. Gelation occurred at room temperature after about 20 h. The investigated sample was denoted as SiO_2_/15PVP.

### 3.2. Methods for Characterization

X-ray diffraction powder data were collected using a Philips PW1730/10 diffractometer using Ni-filtered CuKα radiation. The scanning rate for crystallinity was 11.2° 2θ/min. SEM images were obtained on a Hitachi S-4100 (Hitachi Ltd., Tokyo, Japan) microscope at an accelerating voltage of 25.0 kV. TEM images were captured using a Hitachi H-600A (Tokyo, Japan). A powdered hybrid specimen was dispersed in a mixture of ethanol and polysorbate 80 (1:1 *v*/*v*) by an ultrasonic generator (intensity of 250 W) for 3 min. One or two drops of the resulting dispersion were dropped on a 300-mesh copper grid (Tedpella, Inc., Redding, CA, USA) coated by carbon film and left to evaporate.

Atomic Force Microscopy (AFM) was used for the topographical contour mapping and roughness analysis of the model nanohybrid. Surfaces were mapped in the tapping mode (Intermittent Contact Mode) using NanoScope IIIa Dimension 3100 (Digital Instruments, Inc., Tokyo, Japan) with a prefabricated cantilever with a scan rate of 1.0 Herz; a nominal tip radius of 5–10 nm; a cantilever length of 125 μm; and a resonant frequency of ~200–400 Hz.

An “Evolution 300” UV–Vis diffused reflectance spectrophotometer, utilizing a magnesium oxide reflectance standard as a baseline, was employed to measure the optical absorption spectra of powdered samples within the wavelength range of 200 to 800 nm. The band gap energies (Eg) of the samples were calculated using Planck’s equation, as already described elsewhere [27,28].

### 3.3. Materials for Antimicrobial Activity Testing

For the purpose of the present experiment, microorganisms provided by the National Bank for Industrial Microorganisms and Cell Cultures (NBIMCC, Sofia, Bulgaria) were used. Two types of opportunistic pathogenic bacteria were used—*Staphylococcus epidermidis* ATCC 14990 (Gram-positive) and *Salmonella typhimurium* ATCC BAA-2162, which is Gram-negative. In addition, two types of yeast were used—*Candida albicans* ATCC 18804, a commensal eukaryotic microorganism, and *Saccharomyces cerevisiae* CCY 21-6-3, a eukaryote widely used in the biotechnological industry. The SiO_2_/15PVP hybrid nanomaterial was tested for antimicrobial efficacy alone and in combination with conventional antibiotics in liquid medium. Antibiotics were selected from five groups with a different mode of action, including Ciprofloxacin, an antibiotic belonging to the fluoroquinolone group. It is a broad-spectrum antibiotic and can be used to treat many bacterial infections. It exhibits activity against Gram-negative bacteria. Also, we used Vancomycin—a glycopeptide antibiotic used for complicated skin infections, bone and joint infections, and meningitis caused by MRSA. Its mechanism of action is aimed at the synthesis of the bacterial cell wall by binding to the basic building block. Nystatin was chosen as an antifungal agent as it is used to treat *Candida* skin infections and esophageal and vaginal candidiasis [29].

The minimum bactericidal concentration (MBC) of the sample alone and in combination with antibiotics against different microorganisms was examined using the broth microdilution method, as described by international protocols [30]. The control sample, used in all experiments, consisted of a pure culture of the strain in the exponential growth phase, with a McFarland OD of 0.5, and showed 10^8^ bacterial colonies or 10^6^ yeast colonies, demonstrating the typical growth of the target microorganism without the application of microbicidal agents—antibiotic and nanoparticles. In all experiments, reporting of results and enumeration of surviving cells occurred 24 h after treatment with nanoparticles, antibiotics, and a combination of both. Preparation of the hybrid nanomaterials included the following process: dispersions of nanocomposites in their respective concentrations (100 mg/mL, 50 mg/mL, and 25 mg/mL), by measuring the dry substance with analytical accuracy and dissolving it in distilled water. The respective antibiotics used for the purpose of the experiment were prepared in the same way.

### 3.4. Methodology for Evaluation of the Minimum Inhibitory and Minimal Bactericidal Concentration (MIC) and Antibacterial Mode of Inhibition of SiO_2_/15PVP

The minimum inhibitory concentration (MIC) of the nanocomposites applied in combination with the antibiotic was determined by applying the microdilution method in a 96-well plate. Before starting the experiment, the respective antibiotic was diluted using a sterile 96-well plate. Next, 200 μL of the antibiotic at its initial concentration of 100 μg/mL was instilled into rows A1 and B1 of the 96-well plate. This was followed by a two-fold dilution of the antibiotic by taking 100 μL from the previous well and adding it to each subsequent well until reaching the lowest concentration in wells A11 and B11 of 0.09 μg/mL. When the antibiotic was ready for work, it was mixed with the hybrid material on a new plate: first, 50 μL of the antibiotic in its different concentrations was dripped into one well, and 50 μL of the hybrid material was added to it, again in the corresponding concentration. Finally, 100 μL of bacterial suspension was dripped onto the wells that contained the bactericidal agents. In column 12 of the 96-well plate, only the bacterial suspension was added, which serves as a control. The bacterial growth from the 96-well plate suspensions was monitored by preparing tenfold dilutions by dropping 100 µL onto solid agar and rubbing it until dry. The Petri dishes were incubated for 24 h, at the appropriate temperature, specific to each tested microorganism, and the resulting colonies from the respective dilution were counted. The amount of surviving treated microorganisms was determined by the following formula: CFU/mL (number of colonies × dilution factor)/inoculum sample volume. The minimum bactericidal concentration (MBC) demonstrates the lowest level of antimicrobial agent that results in microbial death. The MBC is identified by determining the lowest concentration of antibacterial agent that reduces the viability of the initial bacterial inoculum by ≥99.9% [31]. Experiments were performed in three independent replicates, and values from each replicate were averaged.

### 3.5. Chemiluminescence Assay

#### 3.5.1. Materials

We purchased the following compounds at high purity: iron sulfate (p.a.) (Merck, Darmstadt, Germany), hydrogen peroxide (30%) (Merck, Darmstadt, Germany), lucigenin (bis-*N*-methylacridinium nitrate) (p.a.) (Sigma-Aldrich, St. Louis, MO, USA), dimethyl sulfoxide (p.a.) (DMSO, Sigma-Aldrich, St. Louis, MO, USA), and buffer pH 7.4 (Sigma-Aldrich, St. Louis, MO, USA). All chemicals were used as purchased.

#### 3.5.2. Method

The chemical probe that we applied for signal amplification is lucigenin. Thus, reliable, comparable differences were achieved.

Fenton’s model system generates ·OOH and ·OH radicals [12]. The control samples do not contain the tested material (which was applied in final concentrations of 1 or 2 mg/mL). The reactions were monitored for 3 min, every 3 s, measured in triplicate, and presented as average and standard deviation values. All tested materials were sonicated for 30 min prior to testing to ensure good dispersion.

Fenton’s system

(1) Fe^2+^ + H_2_O_2_ → Fe^3+^ + ·OH + ^−^OH
(2) Fe^3+^ + H_2_O_2_ → Fe^2+^ + ·OOH + H^+^

#### 3.5.3. Statistics

All experiments were performed using LUMIstar Omega (BMG Labtech GmbH, Ortenberg, Germany, 2020) in triple-reproducible measurements; statistical analysis was performed with OriginPro 8 and Microsoft Office Excel 2010.

### 3.6. Daphnia Magna Toxicity Testing

An acute toxicity test of the resulting SiO_2_/15PVP hybrid was conducted on *Daphnia magna*, with the aim of predicting the maximum permissible concentration in the environment without harm to aquatic organisms. The experiments used 4 concentrations of the substance (0.01 mg/mL; 0.005 mg/mL; 0.001 mg/mL; 0.0001 mg/mL) with 3 replicates of each concentration and 2 controls. The results were detected at 48 h and processed statistically with the Excel program.

## 4. Conclusions

The synthesis of hybrid nanocomposites containing TMOS and poly (vinylpyrrolidone) was carried out via the sol–gel method at room temperature. The morphological structure of the final product was determined by the incorporated PVP. The observed UV–Vis analysis results showed that the obtained hybrid exhibited absorption in the ultraviolet range.

The as-prepared SiO_2_/15PVP hybrid demonstrated a bacteriostatic effect, but its efficacy decreased with decreasing concentration. This was evidenced by the higher number of surviving microorganisms at lower concentrations of SiO_2_/15PVP. The antibiotics Vancomycin and Ciprofloxacin were significantly more effective when applied in combination with SiO_2_/15PVP in inhibiting the growth of *Staphylococcus epidermidis* ATCC 14990 and *Salmonella typhimurium* ATCC BAA-2162, at all antibiotic concentrations tested. The action of Nystatin was significantly enhanced when it was administered in combination with the hybrid materials, especially at a concentration of 100 mg/mL of SiO_2_/15PVP.

The registered and confirmed prooxidant activity of SiO_2_/15PVP shows possible applications at very low concentrations. A significant, nearly threefold increase in the effect of the newly synthesized material at 2 mg/mL in comparison to 1 mg/mL was observed.

*Daphnia magna* was more sensitive than the bacteria to the tested nanoparticles. The harmless concentration to the environment is 0.01 mg/mL according to the conducted experiments.

The obtained results demonstrate the possibility of various clinical implementations of the new synthesized hybrid material.

## Figures and Tables

**Table 1:** Cut-off and optical band gap values for the investigated materials.

Sample	Cut-Off, nm	Eg, eV
PVP	267.77	4.63
TMOS	228.16	5.43
SiO_2_/15PVP	237.61	5.22

## Data Availability

Data are contained within the article.
